# Synergized immunization programming: Pakistan’s road to polio eradication

**DOI:** 10.7189/jogh.11.03103

**Published:** 2021-09-04

**Authors:** Zaeem ul Haq, Muhammad Safdar Rana

**Affiliations:** 1 Expanded Program on Immunization, Islamabad, Pakistan; 2 Ministry of National Health Services, Regulations & Coordination, Islamabad, Pakistan

## THE SITUATION

Vaccines are one of the most powerful biological weapons against disease; the success of COVID-19 vaccination is proof yet again. Countries like the UK that were seeing up to 60 000 new cases and 1218 deaths every day in January 2021, have had a COVID-19 related death rate of less than 100 per day in August 2021 despite having 50 000 new cases every day. The differential between the two time points is brought about by the COVID-19 vaccine; 60% of UK population is fully covered [1]. Ironically, however, there are infections for which vaccines have been available for decades, but the diseases have their debilitating presence, even today. Poliomyelitis is a case in point.

Pakistan, one of the two countries where polio persists, was close to polio eradication in 2017 with eight Wild Polio Virus 1 (WPV1) cases. Unfortunately, the situation took a downturn with WPV1 cases rising to 12 in 2018, and 147 in 2019. In 2019, Pakistan also had an outbreak of circulating Vaccine Derived Polio Virus 2 (cVDPV2) with 22 reported cases. After a brief pause in polio campaigns because of COVID-19, Pakistan resumed its activities in August 2020. Owing to the relentless efforts, the WPV1 cases were 84 in 2020, while only one has been reported during 2021 till the end of May. Similarly, the 135 cVDPV2 cases during 2020 have come down to seven in 2021 till the submission of this report [2].

In countries like Pakistan, where the Polio Eradication Initiative (PEI) and Expanded Program on Immunization (EPI) exist in parallel, an intriguing relationship is observed between the two workstreams. Population clusters with polio cases mostly have a large number of unimmunized or under-immunized children, and consequently, the risk is high for continued circulation and outbreaks [3]. According to PEI’s National Emergency Action Plan (NEAP), out of the 147 WPV1 cases reported in 2019 from Pakistan, 93 (63%) were zero-dose, ie, they had received no vaccination; a proof of the weak routine immunization [4].

The most recent EPI Figures [5] show that about 70% children receive full vaccination, which means that a whopping one third of Pakistani children are still not fully immunized. It is this group of uncovered children, which if not immunized, can lead to an addition in polio cases, and jeopardize the success of complete polio eradication. Important in this context are the regional disparities, usually masked by national figures. Within the overall national picture of 70% is the province of Balochistan where only 27% children have received full immunization [5]. The Quetta block from the same province is one of the three super high-risk areas where polio persists [4].

Pakistan needs to improve its Routine Immunization (RI) to interrupt the WPV along with mainstreaming polio into EPI and maintaining the polio-free status. The continued joint working of the two programs is paramount [6], just as it has been during the current pandemic. Under one-leadership, the PEI surveillance system became the backbone for COVID-19 response, while EPI became the supply line of the vaccine. Similar synergies are required for a polio-free Pakistan. However, there is a need for unpacking the concept of synergy and facilitating its implementation.

## THE CHALLENGE

The issue of zero-dose children, highlighted by NEAP [4], is at the heart of the discussion on Synergy. The lists of these children, documented by polio workers as children with no vaccination, are shared with EPI. The EPI workers, however, neither agree to the definition used for zero-dose nor get the full information about these children for proper response in the field. In addition, whatever coverage they achieve of these zero-dose children, is not shared back with PEI. As a result, there is confusion about the real coverage data of these zero-dose children.

**Figure:**
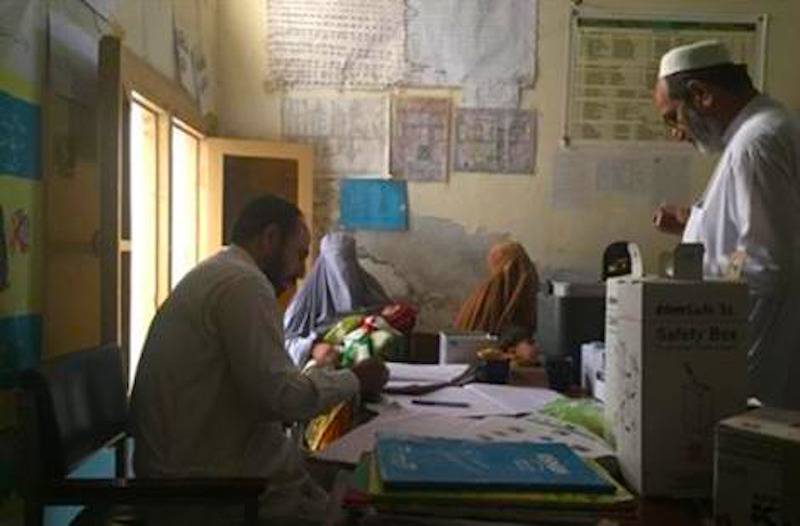
Photo: EPI workers vaccinating children at a vaccination centre in Peshawar, Pakistan (Photo credit: Ayesha Naeem, used with permission).

The administration of birth dose is another significant issue for both programs. The role of polio vaccine at the time of birth is advocated especially in countries where early induction of immunity is imperative [7]. In Pakistan, over 66% of babies are born in a health facility, 44% of them at a private center [8]. It is ironic that because of less coordination between preventive and curative arms of the health system, many among the 66% born in a health facility are sent home without the birth dose, only to be traced again for this purpose. The remaining 34% born mostly at home, are an additional challenge, that needs supplementary effort.

The global guidance like the Polio Endgame Strategy 2018-23, and the Gavi 5.0 strategy underscore the strengthening of RI for achieving the immunization targets [3,9]. The Polio Endgame Strategy emphasizes improved integration during the Supplementary Immunization Activities (SIAs). it elaborates that for expanded partnerships, PEI will collaborate within and beyond the health sector (eg, health, nutrition, and water, sanitation, and hygiene) partners and programs. The Gavi 5.0 Strategy as part of its goal 2 on equity mentions covering the zero-dose children. Both documents do not dwell on the details of the integration of two programs at various levels, importantly the field level [3,9].

The local guidance is reflected in the NEAP of the PEI and several program documents of EPI. The current NEAP [4] identifies the synergy between PEI and EPI, and the Integrated Service Delivery (ISD) among the five main areas of work. However, it stops at suggesting the synchronization of outreach activities, child health days, and integrated campaigns, without elaborating on how these will address the primary issue. The guidance for EPI is fragmented between several project streams and their respective targets and indicators. One of them, the Gavi Health System Strengthening includes covering the zero-dose children and implementing synergized activities with PEI, but the plans are yet to see fruition.

A few years back, our research [10] also unraveled a “stated” synergy from both sides. For EPI, the procurement and logistics for polio vaccine, and the assistance provided by its workers during the SIAs were ‘synergy’. Likewise, the PEI staff said they share the zero-dose data, as well as provide communication and social mobilization assistance to EPI. Deeper discussions, however, revealed an underlying perception on either side that the ‘other’ program is responsible for their below-par performance. The independent experts, during the same discussions, described the two as unequal programs- a disproportion that ought to be addressed to ensure synergy [10].

## THE SOLUTION

**Figure 1:**
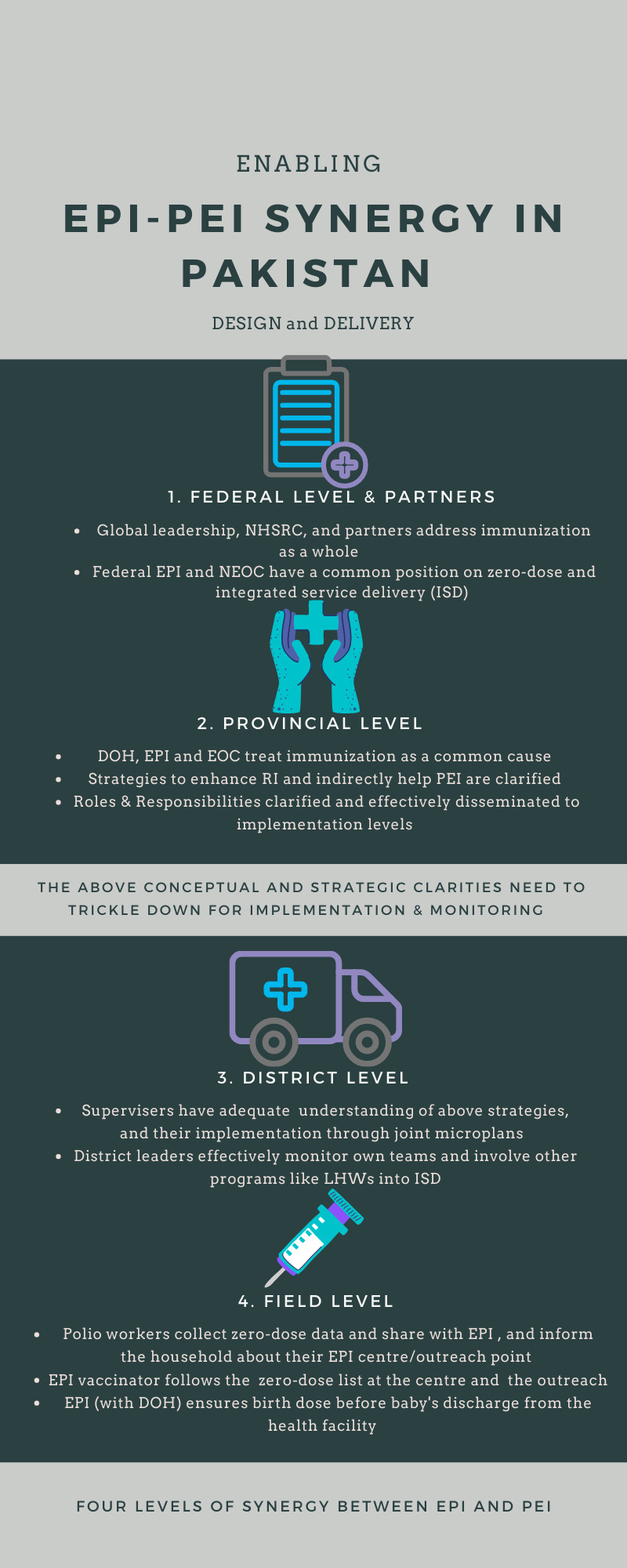
The four levels of synergy between Expanded Program on Immunization and Polio Eradication Initiative in Pakistan.

The synergy between EPI and PEI, which is crucial for achieving and sustaining polio-free Pakistan, and improving routine immunization (RI) as well, requires unpacking the concept at the federal and provincial level, with its operationalization ensured at the district and field level (**Figure 1**). The shared vision of *achieving a polio-free Pakistan through improved RI* needs to be adopted at all levels. The adoption should be incorporated into strategic plans, along with monitoring and evaluation.

The issue of immunizing the zero-dose children must be prioritized. The 10 000 EPI vaccinators have the vaccine but not the access to community and households, which over 250 000 polio staff have, across the country. The polio worker, on the other hand, has access but not the means for reaching the goal of universal immunization. This requires that not only the two programs at federal and provincial levels agree to a common strategy, the same consensus also exists at the level of the Ministry of Health and its global partners.

The following may be considered:

1. Polio workers collect zero-dose data and share it with EPI in a way that the EPI system can reach these children in their community. At the same time, the polio workers, while documenting these zero-dose children, inform the family about the vaccinators working in their union council, and the vaccinator’s schedule at the health center as well as the outreach point.
2. The EPI vaccinator follows the zero-dose list at the health center as well as during outreach to identify and cover all the zero-dose children. After covering these children, the vaccinator shares back the data with polio workers so that the same children are not included again in the list of missed children.

The issue of birth dose requires EPI to ensure a decision by the provincial Department of Health (DoH) that all health facilities providing maternity services must have a vaccination facility, and a newborn should not be discharged before the birth dose. For children born at home, community-based health providers like Community Midwife (CMW) and Traditional Birth Attendants (TBA) must be involved. Following may be considered:

1. A large proportion of children are born in hospitals located in urban centers. The district and tehsil-level hospitals, as well as private nursing homes, must be targeted for the provision of mandatory birth dose before a newborn is discharged. Since big hospitals have huge turnover and mothers having a normal birth are discharged quickly, the vaccination service should be 24/7 so that no child is missed.
2. For babies delivered at home, EPI needs a strong partnership with other vertical programs like Lady Health Worker (LHW) and Maternal, Newborn, Child Health (MNCH) so that CMWs and TBAs can be engaged in facilitating the birth dose of babies delivered by them.

Integrating immunization with other health services, and addressing the community concerns of their overall health needs has to be part of both the intervention streams, mentioned above.
